# Account of an Extraordinary Case of Disease in the Stomach; with the Dissection of the Body after Death

**Published:** 1808-02

**Authors:** Edward D. Smith


*Account of an extraordinary Case of Disease in the Stomach; with the Dissection of the Body after Death. By* Dr. Edward D. Smith.

ON December 13th, 1802, I. E. a house-carpenter, applied to Dr. S. for advice in his case. His appearance was that of one, who had been reduced by long struggles with chronic disease; countenance sallow, and limbs somewhat emaciated. He complained of a constant, and sometimes acute, pain in the lower part of the abdomen, towards the left side. Also of a very disordered state of his stomach and bowels, labouring under want of appetite, and all the other symptoms of dyspepsia and continual constipation. In the part affected, there appeared to be a considerable tumour of a schirrous hardness, which, from its proximity to the situation of the spleen, might possibly be that viscus in an indurated state. The preceding history was as follows. He had laboured under general indisposition in the spring of the year, and this affection was subsequent to his indisposition. Travelling through the northern states during the summer, the pain in his side became so vehement, that he was compelled to seek medical assistance in the town of Providence, state of Rhode-Island. By the advice of two physicians, who supposed the tumour to originate from an affection of the spleen, he was put upon a mercurial course, and a blistering plaster was applied to the place. The plaster had the strange effect of causing a delirium so violent, that it was obliged to be speedily removed. The use of mercury was continued until he was completely salivated. No material permanent relief was obtained; dyspeptic symptoms began to harass him much; his strength and flesh decreased; and he returned, in this situation, to Charleston.

The situation of the tumour induced the belief, that no other other viscus than the spleen could be affected; and notwithstanding the little success resulting from the remedies already employed, it was determined that an effort should be made for the relief of what was deemed the primary affection; and that suitable remedies should, at the same time, be administered for that, which was secondary or symptomatic, now become almost as urgent as the other.

As some idiosyncrasy appeared to forbid the application of cantharides, in their stead the emp. stomachic. was ordered. A strict attention to the usual diet in dyspepsia was enjoined; and the use of garlic, as a tonic to the stomach, was directed. To alleviate a troublesome flatulency then present, a mixture of ol. anis. and bals. capivi was prescribed. For several days the patient thought himself much relieved from all the symptoms, and was able to walk about with tolerable ease. On the night of the 18th, he was seized with violent pain in the tumour, which was somewhat relieved by venæsection.—Now confined to his bed, and suffering intense local pain, he was again urged to submit to vesication, and consented. No such effect followed the application as formerly. The pain continued constant and severe, and the tumour appeared to be more in the front of the abdomen. At the request of the patient, Dr. R. was called into consultation. The emp. calid. was applied over the surface of the blistered part; anodynes were prescribed at night, and purgatives during the day. A similarity was supposed to exist between this case, and a remarkable disease of the bowels recorded by Morgagni. The symptoms had become exceedingly distressing. The stomach rejected almost every thing taken into it, the bowels were obstinately costive, and no natural rest could be procured; a pulsation was now evident in the tumour, and appearances seemed to indicate the formation of an abscess in the abdominal muscles.

The tumour even shewed a disposition to point; cold external applications were first made, and afterwards warm, but it remained in the same state. By desire of Mr. E.'s friends, Dr. W. was requested to attend in consultation. Dr. W. had visited him in a prior indisposition, which, it was supposed, might have some connection with this, and gave the following account of it:

In the latter end of October, 1801, Mr. E. after having supped out, on returning home found the gate of the lot, in which he lived, locked. It was of a considerable height, and he attempted to jump over it, by placing his hands upon the top, and clambering up with his feet; losing his hold, hold, he came with much force upon the ground, and felt (as he said) as if something within him had given way. In consequence of the fall, a swelling of the testicle took place, accompanied with great pain, &c. The antiphlogistic treatment was pursued with him, his complaint proving obstinate, although not to the extent of salivation. He finally gained relief, and was not much harassed again, until the time already mentioned. The idea was now suggested, that the tumour might be an aneurism of some of the large vessels in the abdomen, produced by the violence of the fall.

Under this supposition the case presented a hopeless aspect, and medicine could do nothing but palliate the miseries of the sufferer. His stomach and bowels continued in such condition, that cathartics and anodynes were indispensably necessary to his existence. To these the use of the warm bath was added, until he became so weak, that he was no longer able to bear the fatigue of going to it. Mercury was used for a few days, both internally and by friction; but did not afford any sensible relief. The symptoms daily became more distressing; little or no nourishment could be retained on the stomach; and general emaciation seemed to promise a speedy termination of distress. The patient lingered, in this condition, until February 19th, 1803; when the vital powers, exhausted with the struggle, quietly gave up the conflict. It was very desirable to obtain satisfactory information of the true nature of this case, by examination after death. Permission being obtained from the friends, Dr. W. and myself opened the body, in the presence of several physicians, when the following appearances presented themselves.

### *Dissection.*

The cavity of the abdomen being opened, a tumour was observed in the upper part of it; which, upon examination, appeared to be seated in the stomach. Only a small portion of the stomach could be brought into view, that portion, which is covered by the liver, adhering so firmly, that it was obliged to be separated by the knife. The stomach being dissected away, was removed from the abdomen; very little of the omentum remained in its natural situation, so that its absence from its usual position excited the supposition, that the tumour was composed of it, until more accurate investigation ascertained its seat. The mesenteric glands were enlarged and indurated. The intestines intestines preserved their natural situation and appearance; but were full of scybala.

The liver, kidneys, &c. presented no unusual appearance. The thorax was examined, but no disorder could be traced in any of its viscera. The large vessels, proceeding from the heart, were collapsed and natural. Upon opening the tumour, fæces were discovered in the lower part of it; probably owing to an inverted peristaltic motion. The pylorus was much contracted in size, and discoloured. The different coats of the stomach were obliterated, and presented a disagreeable appearance to the eye. The texture of the viscus was hard and gristly, and the cavity was contracted to one-fourth of its natural size.

Without presuming to offer any comments upon this extraordinary case, I have endeavoured to present a faithful detail, and claim indulgence for the errors of misconception or memory.

*Charleston, May 2d,* 1803.

